# Impact of sphingomyelin levels on coronary heart disease and left ventricular systolic function in humans

**DOI:** 10.1186/1743-7075-8-25

**Published:** 2011-04-26

**Authors:** Xueying Chen, Aijun Sun, Yunzeng Zou, Junbo Ge, Jason M Lazar, Xian-Cheng Jiang

**Affiliations:** 1 Institute of Cardiology, Zhongshan Hospital, Fudan University, Shanghai, PR China; 2 Division of Cardiovascular Medicine, SUNY Downstate Medical Center, Brooklyn, NY, USA; 3 Department of Cell Biology, SUNY Downstate Medical Center, Brooklyn, NY, USA

**Keywords:** sphingomyelin, left ventricular ejection fraction, coronary heart disease, lipids

## Abstract

Sphingomyelin (SM) is an abundant phospholipid in cell membranes and in lipoproteins. In human plasma, SM is mainly found in atherogenic lipoproteins; therefore, higher levels of SM may promote atherogenesis. We investigated the relations between plasma SM levels and the presence of angiographic coronary heart disease (CHD) and left ventricular systolic dysfunction. We studied 732 patients referred for coronary angiography. Median SM levels were higher among patients with CHD and in those with LV systolic dysfunction (LVEF<50%) than in patients without CHD or LV dysfunction. SM levels were significantly correlated with fibrinogen levels, diabetes, apoB, and triglyceride levels. On multivariate analyses, higher median SM levels were associated with a higher risk of CHD and lower LV ejection fraction. The pro-atherogenic property of plasma SM might be related to 1) CHD; 2) LV systolic dysfunction; and 3) metabolism of apoB-containing or triglyceride-rich lipoproteins.

## Introduction

Sphingomyelin (SM), the second most abundant phospholipid in mammalian plasma, is present in all major lipoproteins. Up to 18% of total plasma phospholipid exists as SM [1], with the ratio of phosphatidylcholine (PC)/SM varying widely among lipoprotein subclasses [2]. Atherogenic lipoproteins such as VLDL and LDL are SM-enriched [1,3]. The SM content of atherosclerotic lesions is higher than that of normal arterial tissue [4].

Williams and Tabas have suggested that subendothelial retention and aggregation of atherogenic lipoproteins play an important role in atherogenesis [5,6]. SM-rich LDL retained in atherosclerotic lesions is acted on by an arterial wall sphingomyelinase that appears to promote aggregation, initiating the early phase of atherosclerosis development [7,8]. We have previously found that plasma SM levels in ApoE KO mice are 4-fold higher than those in WT mice [9], and this may partially explain the increased atherosclerosis found in these animals [10]. Our laboratory and others have also discovered that inhibition of SM biosynthesis significantly decreases plasma SM levels, thus lessening atherosclerotic lesions in ApoE KO mice [11,12].

In case-control studies by Jiang et al [13] and Schlitt et al [14], subjects with coronary heart disease (CHD) had higher plasma SM levels than control subjects. In the multi-ethnic study including 6814 subjects without clinical CHD at baseline by Nelson et al, more extensive subclinical atherosclerosis (carotid intimal-medial wall thickness, ankle-arm blood pressure index, and Agatston coronary artery calcium score) was associated with high plasma SM levels[15]. However, in the same population, Yeboah et al found plasma SM levels to be not predictive of incident CHD events after 5 years of follow-up [16]. Therefore, the question as to whether plasma SM is risk factor for CHD remains controversial. In this study, we investigated potential associations between SM and the presence of angiographic coronary heart disease (CHD) in a Chinese cohort.

## Methods

Study Population: A total of 732 subjects who underwent diagnostic coronary angiography for chest pain in Zhongshan Hospital between January, 2004 and December, 2005 were included in this study. Cardiovascular risk factors/diseases were obtained by clinical history. Coronary heart disease (CHD) (n = 489) was predefined as the presence of a luminal diameter stenosis ≥50% in at least 1 major coronary artery territory (left anterior descending, left circumflex or right coronary artery or their major branches). Among CHD patients, 388 had stable angina pectoris (stable AP) and 101 had acute coronary syndrome (ACS). Non-CHD included subjects who had <50% stenosis in all 3 coronary artery territories (n = 243). Exclusion criteria were evidence of significant concomitant cardiac and non-cardiac disease including severe valvular heart disease, prior known cardiomyopathy, malignancy, or febrile condition. Diabetes mellitus was diagnosed by clinical history, use of hypoglycemic medications or a fasting blood sugar level >7.0 mmol/L (125 mg/dL); hypertension was defined in patients receiving antihypertensive treatment or with known diagnosis of hypertension (blood pressure ≥140/90 mmHg). The study was approved by the Institutional Review Board of Zhongshan Hospital, Fudan University.

Laboratory Evaluation: The laboratory evaluation was performed as described previously (17). Subjects were instructed to fast for at least 8 hours prior to blood sampling. Lipid profile was determined with Hitachi 7600 biochemistry autoanalyzer. Triglyceride (TG), total cholesterol (TC) and high-density lipoprotein-cholesterol (HDL-C) were measured with enzymatic methods (TG, Shanghai Kehuadongling Diagnostics Co, Ltd; TC, Shanghai Kehuadongling Diagnostics Co, Ltd; HDL-C, PEG-modified enzyme HDL-C assay, Kyowa Medex Co). Low-density lipoprotein-cholesterol (LDL-C) was calculated according to the Friedewald formula. ApoA-I, apoE, apoB, and Lipoprotein(a) [Lp(a)] were determined by immunoturbidimetric assays (apoA-I, apoE, and apoB, DiaSyA Diagnostics; Lp(a), Nittobo Boseki Co Ltd).

Plasma SM measurement was performed as described previously[17]. There were 4 steps to the enzymatic measurement of plasma SM levels: 1) bacterial SMase hydrolyzed SM to phosphorylcholine and n-acylsphingosine; 2) alkaline phosphatase generated choline from phosphorylcholine; 3) choline was then used to generate hydrogen peroxide in a reaction catalyzed by choline oxidase; and 4) lastly, hydrogen peroxide was used together with DAOS, 4-aminoantipyrine, and peroxidase, as a catalyst, to generate a blue dye with an optimal absorption at 595 nm. The reaction buffer consisted of 0.05 M Tris-HCl with 0.66 mM calcium chloride, pH 8. The following enzyme concentrations present in a 50 ml reaction buffer: 25 units of SMase, 500 units of alkaline phosphatase, 25 units of choline oxidase, and 1,000 units of peroxidase. DAOS concentration was 0.73 mM, and 4-aminoantipyrine concentration was 0.73 mM. Five microliters of plasma was added to a 100 μl reaction buffer containing enzymes, and after 45 min of incubation at 37°C, the absorption was measured at 595 nm on a spectrophotometric plate reader. The developed color remained constant after the incubation time. Standard SM solution (50 mg/dl) preparation (5 mg of SM) was dissolved in 10 ml of 2% Triton X-100 ethanol solution. Each sample was measured in triplicate and the mean value was presented.

### Statistical Methods

Continuous variables were reported as median and 25-75^th ^interquartile ranges if they were not normally distributed. The distributions for plasma concentrations of SM were skewed (p < 0.001) on both the Kolmogorov-Smirnova and Shapiro-Wilks tests. Accordingly, SM levels are reported as median and 25-75^th ^percentiles. For univariate analyses, Spearman's correlation was used to determine correlations of SM with various atherosclerotic markers. SM levels were also compared among the CHD and non-CHD groups, among patients with and without diabetes, and among the normal LV function and LV dysfunction groups using the non-parametric Kruskal Wallis test. Forced logistic regression was used to determine the relation between SM levels and the presence of CHD using CHD as the dependent variable and SM levels, age, gender, BMI, left ventricular ejection fraction (LVEF), diabetes, smoking, HDL, TC, LDL, TG and hypertension as independent variables. The same independent variables were entered into a forced logistic model to identify predictors of LV systolic dysfunction. In addition, linear regression multivariate analysis was used to analyze predictors of LVEF as a continuous variable. Selected interaction effects were tested between the independent variables in the model. All analyses were done using SPSS version 17 analytical software (SPSS Inc., Chicago, IL).

## Results

**Figure 1:**
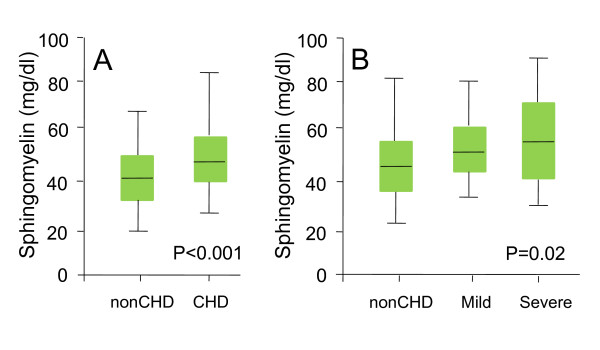
**Median SM levels between CHD and non-CHD patients, and between patients with LV systolic dysfunction (LVEF<50%) and controls (LVEF≥50%)**. Panel A, CHD vs Non-CHD; Panel B, LVEF<50% vs LVEF≥50%.

A total of 732 subjects were included in the study, 489 had CHD. Among the patients with CHD, 101 patients had ACS and 388 had stable AP. Clinical characteristics of the subjects are described in Table 1. Median SM levels were higher among patients with CHD than non-CHD patients (47.2 vs 40.7 mg/dl, p < 0.001) (Figure 1A).

**Table 1:** Group comparisons between Non CHD and CHD as median (25-75 percentiles IQR) or percentage.

**Variable**	**Non CHD**	**CHD**	**P value**
Sphingomyelin(mg/dl)	40.7(33-50)	47.2(39-58)	<0.001
Age(years)	59(53-67)	64(57-71)	<0.001
BMI	24.7(22-26)	24.4(23-26)	1.0
LVEF(%)	69(64-73)	63(55-70)	<0.001
Fibrinogen(mg/dl)	248(212-307)	294(236-356)	<0.001
HDL-C(mmol/L)	1.1(0.9-1.2)	1.0(0.8-1.2)	<0.001
LDL-C(mmol/L)	2.5(2.0-3.0)	2.6(2.1-3.0)	0.37
TC(mmol/L)	4.3(3.7-5.0)	4.3(3.8-4.9)	0.62
TG(mmole/L)	1.5(1.1-2.0)	1.5(1.1-2.2)	0.35
ApoA-I(g/L)	1.1(1.0-1.2)	1.1(0.9-1.2)	0.60
ApoB(g/L)	0.7(0.6-0.9)	0.8(0.6-0.9)	0.03

CHD, coronary heart disease; BMI, body mass index; LVEF, left ventricular ejection fraction; HDL, high density lipoprotein; LDL, low density lipoprotein; TC, total cholesterol; TG, triglyceride; Apo, apolipoprotein.

Of the total 732 patients included in the study, 619 had assessment of LV systolic function. Among those with LVEF, 86.1% had normal LVEF (>50%), 8.6% had mild LV dysfunction (EF ranging between 40-49%), 3.4% had moderate LV dysfunction (EF ranging between 30-39%), and 1.9% had marked LV dysfunction (EF<30%). The moderate and severe groups were grouped together because of the small numbers of patients. Median SM levels were higher among patients with LV systolic dysfunction (LVEF<50%) than in those with normal systolic function (LVEF≥50%) (48.5 vs. 45.5 mg/dl, p = 0.001). As shown in Figure 1B, there was a step-wise increase in median SM levels in patients with normal, mild, and moderate-severe degrees of LV dysfunction (45.5 mg/dL vs. 47.3 mg/dL vs. 57.6 mg/dl, p = 0.021). When considered as a continuous variable, LVEF was significantly correlated with SM levels in all patients (r = -0.20, p < 0.001), in those with CHD (r = -0.15, p = 0.003), and in those without CHD (r = -0.14, p = 0.046).

On univariate analyses, SM levels were significantly correlated with triglyceride levels (r = 0.10, p = 0.005), apoB (r = 0.13, p = 0.001), apoE (r = 0.135, p < 0.001), fibrinogen levels (r = 0.11, p = 0.006), and LVEF (r = -0.201, p < 0.001)(Table 2). There were no significant correlations between SM levels and HDL cholesterol (r = -0.069, p = 0.061), total cholesterol (r = 0.064, p = 0.089), or LDL cholesterol levels (r = 0.035, p = 0.35). Median SM levels were higher for patients with diabetes as compared to those without (47.1 vs. 45.2, p = 0.029).

**Table 2:** Univariate correlations of sphingomyelin.

**Variable**		**Sphingomyelin(mg/dl)**
Fibrinogen	r	0.107
	P	0.006
LVEF	r	-0.201
	P	<0.001
ApoB	r	0.126
	P	<0.001
ApoE	r	0.135
	P	<0.001
TG	r	0.104
	P	0.005

LVEF, left ventricular ejection fraction; TG,

triglyceride; Apo, apolipoprotein.

Using multivariate logistic regression analysis including age, gender, BMI, smoking, hypertension, diabetes, HDL, LDL, TG, LV systolic dysfunction (LVEF<50%) and SM levels, variables that were independently associated with the presence of CHD were: age (p < 0.0001), male gender (p < 0.0001), smoking (p = 0.002), diabetes (p = 0.004), hypertension (p = 0.03), LVEF (p < 0.001) and SM (p < 0.001) (Table 3).

**Table 3:** Logistic regression predictors of CHD.

**Variable**	**P value**	**aOR**	**95% CI**
Sphingomyelin(mg/dl)	<0.001	1.056	(1.04-1.08)
Age(years)	<0.0001	1.062	(1.05-1.09)
Male gender	<0.0001	0.383	(.25-.63)
Smoking	0.002	2.028	(1.3-2.99)
DM(history)	0.004	2.292	(1.66-4.78)
HTN(history)	0.03	1.596	(1.05-2.44)
HDL(mmol/L)	0.071	0.445	(.202-1.10)
TG(mmol/L)	0.085	0.981	(0.90-1.04)
LDL(mmol/L)	0.084	1.210	(.98-1.50)
LVEF(%)	<0.001	0.033	(.005-.212)

CHD, coronary heart disease; DM, diabetes; HTN, hypertension; TG, triglyceride; HDL, high density lipoprotein; LDL, low density lipoprotein; LVEF, left ventricular ejection fraction.

Using multivariate linear regression including the same variables to predict LVEF as a continuous variable, LVEF was independently related to SM levels (p = 0.035) and a trend towards the presence of CHD (p = 0.052) (Table 4).

**Table 4:** Logistic regression predictors of LV systolic dysfunction.

**Variable**	**P value**	**aOR**	**95%CI**
Sphingomyelin(mg/dl)	0.035	1.02	(1.00-1.03)
Age(years)	0.885	0.99	(0.97-1.02)
Male gender	0.074	0.534	(.27-1.06)
Smoking	0.143	0.684	(.41-1.13)
DM(history)	0.618	1.160	(.65-2.07)
HTN(history)	0.309	0.772	(.47-1.27)
BMI	0.107	1.851	(.99-3.44)
CHD	0.052	0.540	(.29-1.00)

DM, diabetes; HTN, hypertension; BMI, body mass index; CHD, coronary heart disease.

## Discussion

In the present study, we found that SM levels were independently associated with CHD, which was in line with our previous observation [13]. Furthermore, we reveal that plasma SM levels were correlated with LV systolic dysfunction, and with plasma apoB and triglycerides levels.

It is known that atherosclerosis is an inflammatory disease. Inflammatory cells such as macrophages and lymphocytes and inflammatory factors including cytokines, chemokines and growth factors, promote focal necrosis and advanced complicated lesion formation. SM might act as a marker for an inflammatory effect in the development and progression of CHD. Our sphingomyelin syntase (SMS) 2 mouse studies indicated that SM biosynthesis is responsible for NF-κB and MAP kinase-mediated inflammatory and atherosclerosis [18,19]. We found that SMS2 deficiency substantially diminished the abundance of toll like receptor 4 (TLR4)-MD2 complex levels on the surface of macrophages after LPS stimulation [18]. Moreover, we found that SMS2 KO mice had less IL-6 and TNFα in the circulation after LPS stimulation, compared with controls [19].

The association between SM levels and LV dysfunction is a novel finding as we are unaware of prior studies reporting such a relation. Although CHD is a leading cause of LV systolic dysfunction, it is unlikely that the SM-LVEF relation is mediated by CHD. SM levels were similarly correlated in an inverse manner with LVEF in both patients with and without CHD. In addition, the relation between SM and LVEF remained significant despite inclusion of CHD in the multivariate model. It remains unclear as to whether higher plasma SM level is a marker of LV dysfunction or whether SM is directly involved in the pathogenesis of LV dysfunction. Alternatively, higher SM levels may represent an adaptive response to LV dysfunction. Inflammation is one of the possible linkages. In our patient population, there was a significant association between SM levels and fibrinogen, an acute phase reactant in inflammation. Prior studies have also found SM metabolism related to levels of CRP[20], inflammatory marker. Inflammation is well known to play a central role in the progression of LV dysfunction and heart failure [21]. Several lines of evidence suggest that SM may play a role in the development of LV dysfunction through promoting inflammation. In cardiomyocytes, activation of the neutral sphingomyelinase, which can hydrolyse SM, mediates TNFα-induced apoptosis and negative contractile effect[22-25]. Sphingomyelinase levels have been shown to correlate with the presence and severity of congestive heart failure [26]. Moreover, Mrnka et al. showed that SM concentration in rat myocytes increased in response to pressure overload, a well known precursor to LV hypertrophy and dysfunction [27]. We previously showed that sphingomyelin synthase 2 (SMS 2), an enzyme directly involving in SM de novo biosynthesis, deficiency reduces plasma and cell membrane SM levels, reduces NFκB-, MAP kinase-mediated inflammatory responses, and thus reduces atherosclerosis in mouse models[28,29]. However, there are few prior data establishing the direct relationship between plasma SM levels and LV dysfunction. This study is the first one. It is quite possible that SM levels, either in the blood or on the plasma membrane of cardio-muscle cells, play an important role in mediating LV dysfunction. The detail mechanism is deserved for further investigations.

In this study, we found the significant correlations between plasma SM and apoB or triglycerides (Table 2). It is well known that SM content is much higher in apoB-containing or triglyceride-rich lipoproteins than in HDL [1]. This may indicate that apoB-containing or triglyceride-rich lipoproteins are atherogenic but that HDL is not. It is also known that non-HDL lipoprotein subendothelial retention is an early step in atherogenesis [30]. It is believed that SM-rich non-HDL lipoproteins retained in atherosclerotic lesions are hydrolyzed by an arterial wall sphingomyelinase that promotes aggregation by converting SM to ceramide [5,31]. We have shown that plasma SM levels can serve as a marker for postprandial lipoprotein particle clearance [32]. If somehow this clearance was blocked, the SM-rich particles could likely be aggregated in the arterial wall after encountering sphingomylinase there, and development or instability of atheroscerotic plaques could be the consequence. Tabas's group provided convincing evidence that apoE KO mice lacking sphingomyelinase have decreased development of early atherosclerotic lesions [8]. We investigated this retention/aggregation event in another angle: reducing SM content of non-HDL lipoproteins through SMS deficient approach, thus leading to less non-HDL lipoprotein retention/aggregation in aorta, and preventing the development of atherosclerosis[33].

This study has a number of limitations. First, the independent relation of SM levels with LV systolic dysfunction should ideally be studied in the context of more established biomarkers of LV systolic dysfunction, such as BNP. Second, given the relatively small number of patients with ACS (n = 101) in our study population, we were unable to perform meaningful subgroup analyses of the relation between SM levels and ACS. Third, the size of the population was relatively small and the study was not designed with a priori calculations with respect to sample size or statistical power. As such, the findings need to be confirmed in larger and prospectively designed studies. Fourth, there is a difference in the age of the two cohorts that were compared. The observed changes in SM may reflect aging-associated differences. Lastly, we used the definition of coronary stenoses as defined>50% by coronary angiography as a crude marker of hemodyanamically significant disease. Therefore, the control group likely did have atherosclerosis. Although numerous studies have used this criterion, quantification of coronary atherosclerosis by intravascular ultrasound is a more sensitive measure of atherosclerotic burden. However, this technique was not performed in the cohort evaluated in the present study.

In conclusion, in this study we found that the pro-atherogenic property of plasma SM might be related to 1) CHD; 2) LV systolic dysfunction; and 3) abnormal metabolism of apoB-containing or triglyceride-rich lipoproteins.

## Consent

Written informed consent was obtained from the patient for publication of this case report and accompanying images. A copy of the written consent is available for review by the Editor-in-Chief of this journal.

## Competing interests

The authors declare that they have no competing interests.

## Authors' contributions

XC and AS carried out the SM measurement and modified the manuscript. YZ and JG provided the samples. JML participated in the design of the study, performed the statistical analysis, and partially drafted the manuscript. XCJ conceived of the study, participated in its design and coordination, and drafted the manuscript. All authors read and approved the final manuscript.
